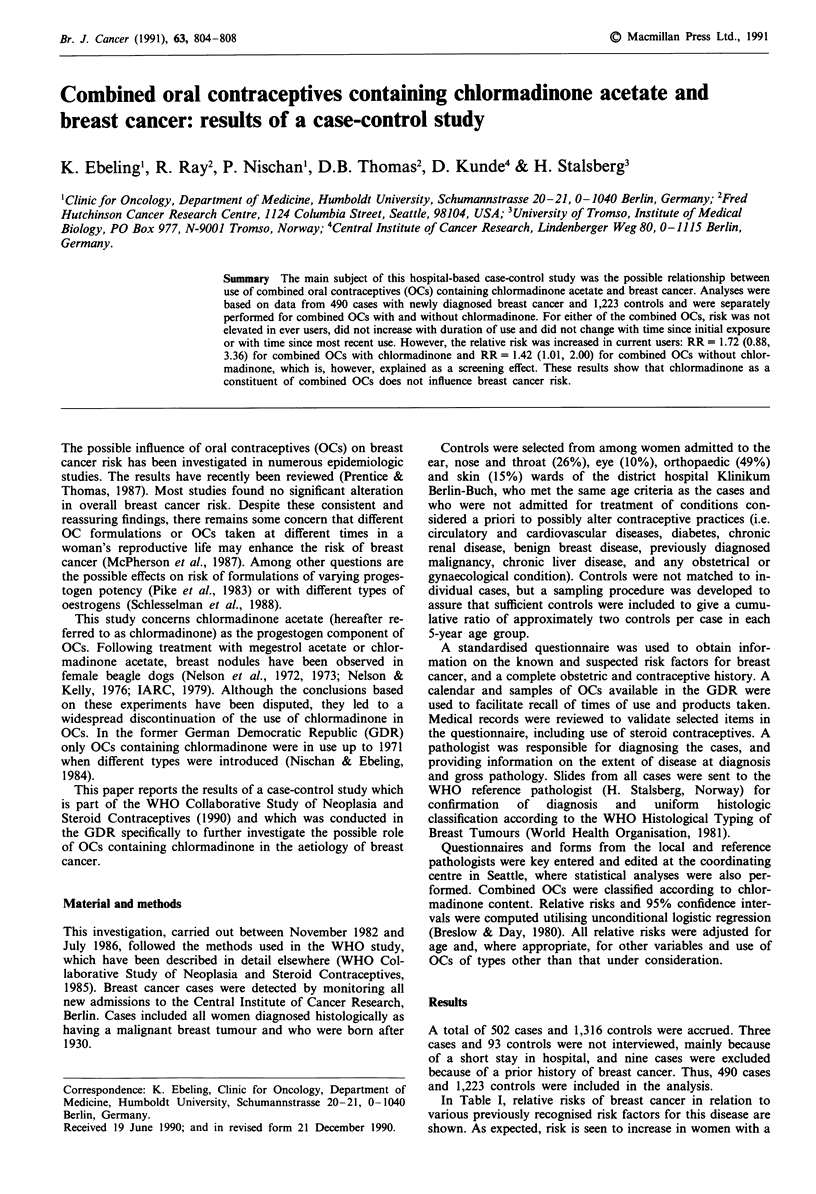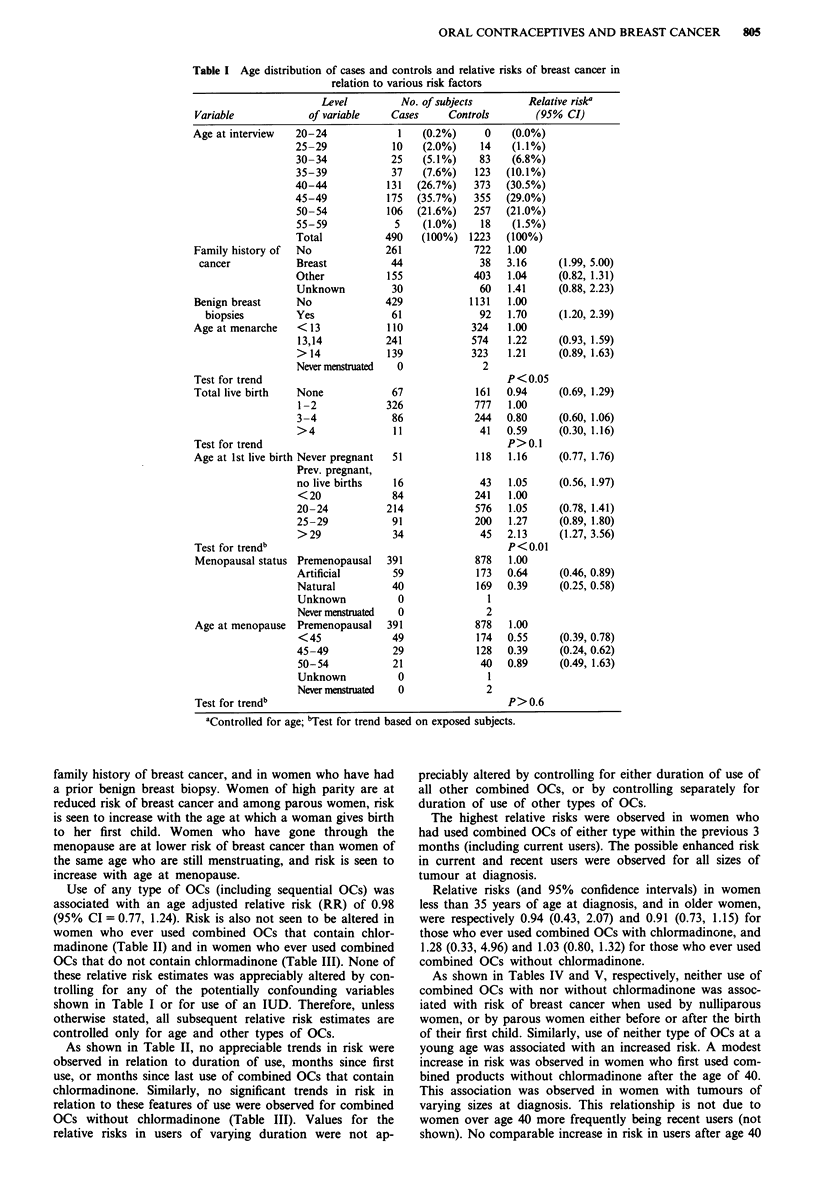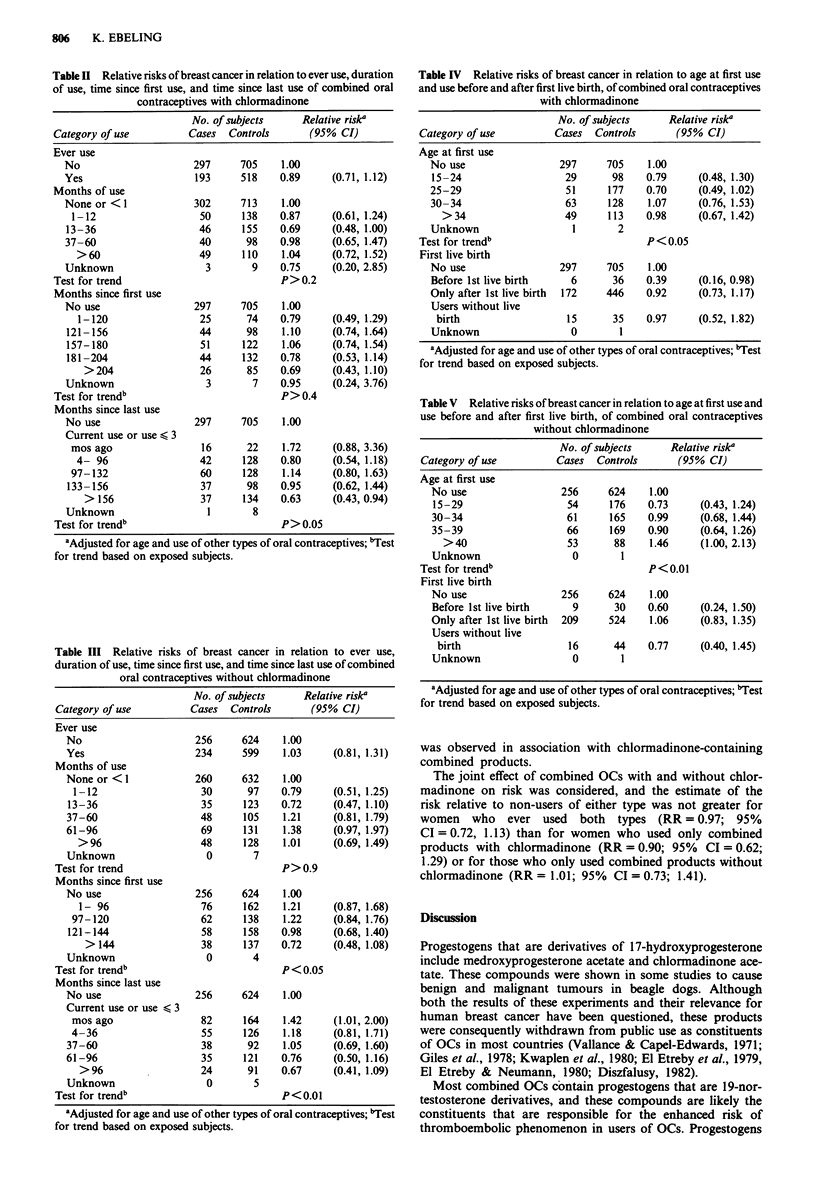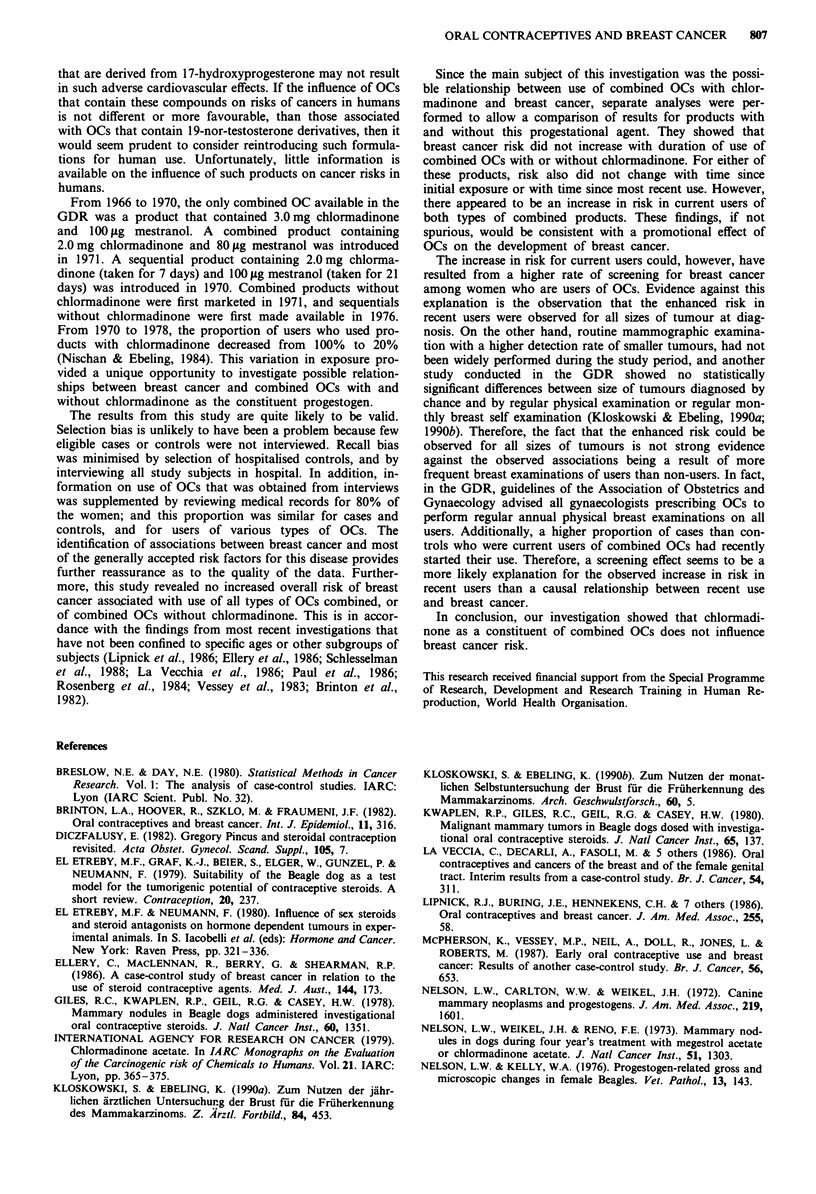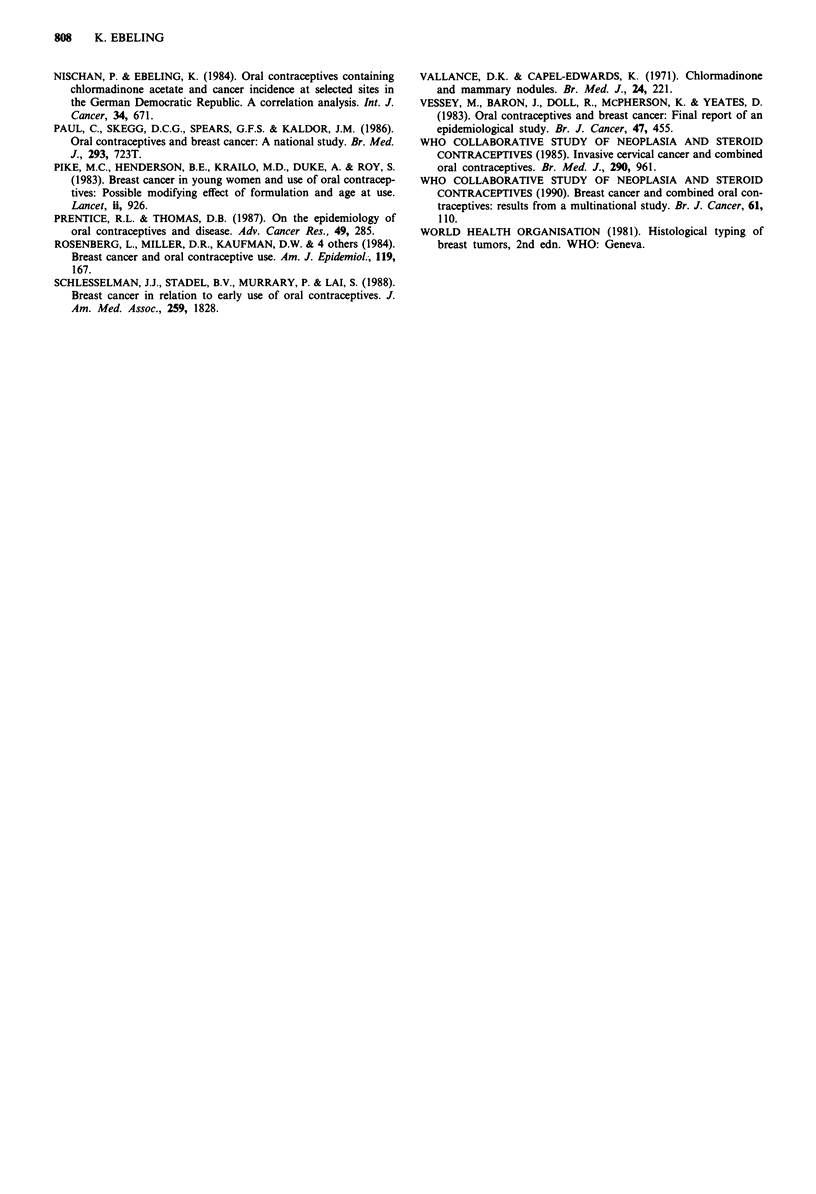# Combined oral contraceptives containing chlormadinone acetate and breast cancer: results of a case-control study.

**DOI:** 10.1038/bjc.1991.178

**Published:** 1991-05

**Authors:** K. Ebeling, R. Ray, P. Nischan, D. B. Thomas, D. Kunde, H. Stalsberg

**Affiliations:** Department of Medicine, Humboldt University, Schumannstrasse Berlin, Germany.

## Abstract

The main subject of this hospital-based case-control study was the possible relationship between use of combined oral contraceptives (OCs) containing chlormadinone acetate and breast cancer. Analyses were based on data from 490 cases with newly diagnosed breast cancer and 1,223 controls and were separately performed for combined OCs with and without chlormadinone. For either of the combined OCs, risk was not elevated in ever users, did not increase with duration of use and did not change with time since initial exposure or with time since most recent use. However, the relative risk was increased in current users: RR = 1.72 (0.88, 3.36) for combined OCs with chlormadinone and RR = 1.42 (1.01, 2.00) for combined OCs without chlormadinone, which is, however, explained as a screening effect. These results show that chlormadinone as a constituent of combined OCs does not influence breast cancer risk.


Br. J. Cancer (1991), 63, 804-808 © Macmillan Press Ltd., 1991

### Combined oral contraceptives containing chlormadinone acetate and breast cancer: results of a case-control study

K. Ebeling[1], R. Ray[2], P. Nischan[1], D.B. Thomas[2], D. Kunde[4] & H. Stalsberg[3]

[1]*Clinic for Oncology, Department of Medicine, Humboldt University, Schumannstrasse 20-21, 0-1040 Berlin, Germany;* [2]*Fred Hutchinson Cancer Research Centre, 1124 Columbia Street, Seattle, 98104, USA;* [3]*University of Tromso, Institute of Medical Biology, PO Box 977, N-9001 Tromso, Norway;* [4]*Central Institute of Cancer Research, Lindenberger Weg 80, 0-1115 Berlin, Germany.*

**Summary** The main subject of this hospital-based case-control study was the possible relationship between use of combined oral contraceptives (OCs) containing chlormadinone acetate and breast cancer. Analyses were based on data from 490 cases with newly diagnosed breast cancer and 1,223 controls and were separately performed for combined OCs with and without chlormadinone. For either of the combined OCs, risk was not elevated in ever users, did not increase with duration of use and did not change with time since initial exposure or with time since most recent use. However, the relative risk was increased in current users: RR = 1.72 (0.88, 3.36) for combined OCs with chlormadinone and RR = 1.42 (1.01, 2.00) for combined OCs without chlormadinone, which is, however, explained as a screening effect. These results show that chlormadinone as a constituent of combined OCs does not influence breast cancer risk.

The possible influence of oral contraceptives (OCs) on breast cancer risk has been investigated in numerous epidemiologic studies. The results have recently been reviewed (Prentice & Thomas, 1987). Most studies found no significant alteration in overall breast cancer risk. Despite these consistent and reassuring findings, there remains some concern that different OC formulations or OCs taken at different times in a woman's reproductive life may enhance the risk of breast cancer (McPherson *et al.*, 1987). Among other questions are the possible effects on risk of formulations of varying progestogen potency (Pike *et al.*, 1983) or with different types of oestrogens (Schlesselman *et al.*, 1988).

This study concerns chlormadinone acetate (hereafter referred to as chlormadinone) as the progestogen component of OCs. Following treatment with megestrol acetate or chlormadinone acetate, breast nodules have been observed in female beagle dogs (Nelson *et al.*, 1972, 1973; Nelson & Kelly, 1976; IARC, 1979). Although the conclusions based on these experiments have been disputed, they led to a widespread discontinuation of the use of chlormadinone in OCs. In the former German Democratic Republic (GDR) only OCs containing chlormadinone were in use up to 1971 when different types were introduced (Nischan & Ebeling, 1984).

This paper reports the results of a case-control study which is part of the WHO Collaborative Study of Neoplasia and Steroid Contraceptives (1990) and which was conducted in the GDR specifically to further investigate the possible role of OCs containing chlormadinone in the aetiology of breast cancer.

### Material and methods

This investigation, carried out between November 1982 and July 1986, followed the methods used in the WHO study, which have been described in detail elsewhere (WHO Collaborative Study of Neoplasia and Steroid Contraceptives, 1985). Breast cancer cases were detected by monitoring all new admissions to the Central Institute of Cancer Research, Berlin. Cases included all women diagnosed histologically as having a malignant breast tumour and who were born after 1930.

Controls were selected from among women admitted to the ear, nose and throat (26%), eye (10%), orthopaedic (49%) and skin (15%) wards of the district hospital Klinikum Berlin-Buch, who met the same age criteria as the cases and who were not admitted for treatment of conditions considered a priori to possibly alter contraceptive practices (i.e. circulatory and cardiovascular diseases, diabetes, chronic renal disease, benign breast disease, previously diagnosed malignancy, chronic liver disease, and any obstetrical or gynaecological condition). Controls were not matched to individual cases, but a sampling procedure was developed to assure that sufficient controls were included to give a cumulative ratio of approximately two controls per case in each 5-year age group.

A standardised questionnaire was used to obtain information on the known and suspected risk factors for breast cancer, and a complete obstetric and contraceptive history. A calendar and samples of OCs available in the GDR were used to facilitate recall of times of use and products taken. Medical records were reviewed to validate selected items in the questionnaire, including use of steroid contraceptives. A pathologist was responsible for diagnosing the cases, and providing information on the extent of disease at diagnosis and gross pathology. Slides from all cases were sent to the WHO reference pathologist (H. Stalsberg, Norway) for confirmation of diagnosis and uniform histologic classification according to the WHO Histological Typing of Breast Tumours (World Health Organisation, 1981).

Questionnaires and forms from the local and reference pathologists were key entered and edited at the coordinating centre in Seattle, where statistical analyses were also performed. Combined OCs were classified according to chlormadinone content. Relative risks and 95% confidence intervals were computed utilising unconditional logistic regression (Breslow & Day, 1980). All relative risks were adjusted for age and, where appropriate, for other variables and use of OCs of types other than that under consideration.

### Results

A total of 502 cases and 1,316 controls were accrued. Three cases and 93 controls were not interviewed, mainly because of a short stay in hospital, and nine cases were excluded because of a prior history of breast cancer. Thus, 490 cases and 1,223 controls were included in the analysis.

In Table I, relative risks of breast cancer in relation to various previously recognised risk factors for this disease are shown. As expected, risk is seen to increase in women with a

Correspondence: K. Ebeling, Clinic for Oncology, Department of Medicine, Humboldt University, Schumannstrasse 20-21, 0-1040 Berlin, Germany.
Received 19 June 1990; and in revised form 21 December 1990.

**Table I** Age distribution of cases and controls and relative risks of breast cancer in relation to various risk factors

| Variable | Level of variable | No. of subjects Cases | | Controls | | Relative risk[a] (95% CI) | |
|---|---|---|---|---|---|---|---|
| Age at interview | 20–24 | 1 | (0.2%) | 0 | (0.0%) | | |
| | 25–29 | 10 | (2.0%) | 14 | (1.1%) | | |
| | 30–34 | 25 | (5.1%) | 83 | (6.8%) | | |
| | 35–39 | 37 | (7.6%) | 123 | (10.1%) | | |
| | 40–44 | 131 | (26.7%) | 373 | (30.5%) | | |
| | 45–49 | 175 | (35.7%) | 355 | (29.0%) | | |
| | 50–54 | 106 | (21.6%) | 257 | (21.0%) | | |
| | 55–59 | 5 | (1.0%) | 18 | (1.5%) | | |
| | Total | 490 | (100%) | 1223 | (100%) | | |
| Family history of cancer | No | 261 | | 722 | | 1.00 | |
| | Breast | 44 | | 38 | | 3.16 | (1.99, 5.00) |
| | Other | 155 | | 403 | | 1.04 | (0.82, 1.31) |
| | Unknown | 30 | | 60 | | 1.41 | (0.88, 2.23) |
| Benign breast biopsies | No | 429 | | 1131 | | 1.00 | |
| | Yes | 61 | | 92 | | 1.70 | (1.20, 2.39) |
| Age at menarche | <13 | 110 | | 324 | | 1.00 | |
| | 13,14 | 241 | | 574 | | 1.22 | (0.93, 1.59) |
| | >14 | 139 | | 323 | | 1.21 | (0.89, 1.63) |
| | Never menstruated | 0 | | 2 | | | |
| Test for trend | | | | | | $P<0.05$ | |
| Total live birth | None | 67 | | 161 | | 0.94 | (0.69, 1.29) |
| | 1–2 | 326 | | 777 | | 1.00 | |
| | 3–4 | 86 | | 244 | | 0.80 | (0.60, 1.06) |
| | >4 | 11 | | 41 | | 0.59 | (0.30, 1.16) |
| Test for trend | | | | | | $P>0.1$ | |
| Age at 1st live birth | Never pregnant | 51 | | 118 | | 1.16 | (0.77, 1.76) |
| | Prev. pregnant, no live births | 16 | | 43 | | 1.05 | (0.56, 1.97) |
| | <20 | 84 | | 241 | | 1.00 | |
| | 20–24 | 214 | | 576 | | 1.05 | (0.78, 1.41) |
| | 25–29 | 91 | | 200 | | 1.27 | (0.89, 1.80) |
| | >29 | 34 | | 45 | | 2.13 | (1.27, 3.56) |
| Test for trend[b] | | | | | | $P<0.01$ | |
| Menopausal status | Premenopausal | 391 | | 878 | | 1.00 | |
| | Artificial | 59 | | 173 | | 0.64 | (0.46, 0.89) |
| | Natural | 40 | | 169 | | 0.39 | (0.25, 0.58) |
| | Unknown | 0 | | 1 | | | |
| | Never menstruated | 0 | | 2 | | | |
| Age at menopause | Premenopausal | 391 | | 878 | | 1.00 | |
| | <45 | 49 | | 174 | | 0.55 | (0.39, 0.78) |
| | 45–49 | 29 | | 128 | | 0.39 | (0.24, 0.62) |
| | 50–54 | 21 | | 40 | | 0.89 | (0.49, 1.63) |
| | Unknown | 0 | | 1 | | | |
| | Never menstruated | 0 | | 2 | | | |
| Test for trend[b] | | | | | | $P>0.6$ | |

[a]Controlled for age; [b]Test for trend based on exposed subjects.

family history of breast cancer, and in women who have had a prior benign breast biopsy. Women of high parity are at reduced risk of breast cancer and among parous women, risk is seen to increase with the age at which a woman gives birth to her first child. Women who have gone through the menopause are at lower risk of breast cancer than women of the same age who are still menstruating, and risk is seen to increase with age at menopause.

Use of any type of OCs (including sequential OCs) was associated with an age adjusted relative risk (RR) of 0.98 (95% CI = 0.77, 1.24). Risk is also not seen to be altered in women who ever used combined OCs that contain chlormadinone (Table II) and in women who ever used combined OCs that do not contain chlormadinone (Table III). None of these relative risk estimates was appreciably altered by controlling for any of the potentially confounding variables shown in Table I or for use of an IUD. Therefore, unless otherwise stated, all subsequent relative risk estimates are controlled only for age and other types of OCs.

As shown in Table II, no appreciable trends in risk were observed in relation to duration of use, months since first use, or months since last use of combined OCs that contain chlormadinone. Similarly, no significant trends in risk in relation to these features of use were observed for combined OCs without chlormadinone (Table III). Values for the relative risks in users of varying duration were not appreciably altered by controlling for either duration of use of all other combined OCs, or by controlling separately for duration of use of other types of OCs.

The highest relative risks were observed in women who had used combined OCs of either type within the previous 3 months (including current users). The possible enhanced risk in current and recent users were observed for all sizes of tumour at diagnosis.

Relative risks (and 95% confidence intervals) in women less than 35 years of age at diagnosis, and in older women, were respectively 0.94 (0.43, 2.07) and 0.91 (0.73, 1.15) for those who ever used combined OCs with chlormadinone, and 1.28 (0.33, 4.96) and 1.03 (0.80, 1.32) for those who ever used combined OCs without chlormadinone.

As shown in Tables IV and V, respectively, neither use of combined OCs with nor without chlormadinone was associated with risk of breast cancer when used by nulliparous women, or by parous women either before or after the birth of their first child. Similarly, use of neither type of OCs at a young age was associated with an increased risk. A modest increase in risk was observed in women who first used combined products without chlormadinone after the age of 40. This association was observed in women with tumours of varying sizes at diagnosis. This relationship is not due to women over age 40 more frequently being recent users (not shown). No comparable increase in risk in users after age 40

**Table II** Relative risks of breast cancer in relation to ever use, duration of use, time since first use, and time since last use of combined oral contraceptives with chlormadinone

| *Category of use* | *No. of subjects* Cases | Controls | *Relative risk*[a] | *(95% CI)* |
|---|---|---|---|---|
| Ever use | | | | |
| No | 297 | 705 | 1.00 | |
| Yes | 193 | 518 | 0.89 | (0.71, 1.12) |
| Months of use | | | | |
| None or <1 | 302 | 713 | 1.00 | |
| 1–12 | 50 | 138 | 0.87 | (0.61, 1.24) |
| 13–36 | 46 | 155 | 0.69 | (0.48, 1.00) |
| 37–60 | 40 | 98 | 0.98 | (0.65, 1.47) |
| >60 | 49 | 110 | 1.04 | (0.72, 1.52) |
| Unknown | 3 | 9 | 0.75 | (0.20, 2.85) |
| Test for trend | | | $P>0.2$ | |
| Months since first use | | | | |
| No use | 297 | 705 | 1.00 | |
| 1–120 | 25 | 74 | 0.79 | (0.49, 1.29) |
| 121–156 | 44 | 98 | 1.10 | (0.74, 1.64) |
| 157–180 | 51 | 122 | 1.06 | (0.74, 1.54) |
| 181–204 | 44 | 132 | 0.78 | (0.53, 1.14) |
| >204 | 26 | 85 | 0.69 | (0.43, 1.10) |
| Unknown | 3 | 7 | 0.95 | (0.24, 3.76) |
| Test for trend[b] | | | $P>0.4$ | |
| Months since last use | | | | |
| No use | 297 | 705 | 1.00 | |
| Current use or use ⩽ 3 mos ago | 16 | 22 | 1.72 | (0.88, 3.36) |
| 4–96 | 42 | 128 | 0.80 | (0.54, 1.18) |
| 97–132 | 60 | 128 | 1.14 | (0.80, 1.63) |
| 133–156 | 37 | 98 | 0.95 | (0.62, 1.44) |
| >156 | 37 | 134 | 0.63 | (0.43, 0.94) |
| Unknown | 1 | 8 | | |
| Test for trend[b] | | | $P>0.05$ | |

[a]Adjusted for age and use of other types of oral contraceptives; [b]Test for trend based on exposed subjects.

**Table III** Relative risks of breast cancer in relation to ever use, duration of use, time since first use, and time since last use of combined oral contraceptives without chlormadinone

| *Category of use* | *No. of subjects* Cases | Controls | *Relative risk*[a] | *(95% CI)* |
|---|---|---|---|---|
| Ever use | | | | |
| No | 256 | 624 | 1.00 | |
| Yes | 234 | 599 | 1.03 | (0.81, 1.31) |
| Months of use | | | | |
| None or <1 | 260 | 632 | 1.00 | |
| 1–12 | 30 | 97 | 0.79 | (0.51, 1.25) |
| 13–36 | 35 | 123 | 0.72 | (0.47, 1.10) |
| 37–60 | 48 | 105 | 1.21 | (0.81, 1.79) |
| 61–96 | 69 | 131 | 1.38 | (0.97, 1.97) |
| >96 | 48 | 128 | 1.01 | (0.69, 1.49) |
| Unknown | 0 | 7 | | |
| Test for trend | | | $P>0.9$ | |
| Months since first use | | | | |
| No use | 256 | 624 | 1.00 | |
| 1–96 | 76 | 162 | 1.21 | (0.87, 1.68) |
| 97–120 | 62 | 138 | 1.22 | (0.84, 1.76) |
| 121–144 | 58 | 158 | 0.98 | (0.68, 1.40) |
| >144 | 38 | 137 | 0.72 | (0.48, 1.08) |
| Unknown | 0 | 4 | | |
| Test for trend[b] | | | $P<0.05$ | |
| Months since last use | | | | |
| No use | 256 | 624 | 1.00 | |
| Current use or use ⩽ 3 mos ago | 82 | 164 | 1.42 | (1.01, 2.00) |
| 4–36 | 55 | 126 | 1.18 | (0.81, 1.71) |
| 37–60 | 38 | 92 | 1.05 | (0.69, 1.60) |
| 61–96 | 35 | 121 | 0.76 | (0.50, 1.16) |
| >96 | 24 | 91 | 0.67 | (0.41, 1.09) |
| Unknown | 0 | 5 | | |
| Test for trend[b] | | | $P<0.01$ | |

[a]Adjusted for age and use of other types of oral contraceptives; [b]Test for trend based on exposed subjects.

**Table IV** Relative risks of breast cancer in relation to age at first use and use before and after first live birth, of combined oral contraceptives with chlormadinone

| *Category of use* | *No. of subjects* Cases | Controls | *Relative risk*[a] | *(95% CI)* |
|---|---|---|---|---|
| Age at first use | | | | |
| No use | 297 | 705 | 1.00 | |
| 15–24 | 29 | 98 | 0.79 | (0.48, 1.30) |
| 25–29 | 51 | 177 | 0.70 | (0.49, 1.02) |
| 30–34 | 63 | 128 | 1.07 | (0.76, 1.53) |
| >34 | 49 | 113 | 0.98 | (0.67, 1.42) |
| Unknown | 1 | 2 | | |
| Test for trend[b] | | | $P<0.05$ | |
| First live birth | | | | |
| No use | 297 | 705 | 1.00 | |
| Before 1st live birth | 6 | 36 | 0.39 | (0.16, 0.98) |
| Only after 1st live birth | 172 | 446 | 0.92 | (0.73, 1.17) |
| Users without live birth | 15 | 35 | 0.97 | (0.52, 1.82) |
| Unknown | 0 | 1 | | |

[a]Adjusted for age and use of other types of oral contraceptives; [b]Test for trend based on exposed subjects.

**Table V** Relative risks of breast cancer in relation to age at first use and use before and after first live birth, of combined oral contraceptives without chlormadinone

| *Category of use* | *No. of subjects* Cases | Controls | *Relative risk*[a] | *(95% CI)* |
|---|---|---|---|---|
| Age at first use | | | | |
| No use | 256 | 624 | 1.00 | |
| 15–29 | 54 | 176 | 0.73 | (0.43, 1.24) |
| 30–34 | 61 | 165 | 0.99 | (0.68, 1.44) |
| 35–39 | 66 | 169 | 0.90 | (0.64, 1.26) |
| >40 | 53 | 88 | 1.46 | (1.00, 2.13) |
| Unknown | 0 | 1 | | |
| Test for trend[b] | | | $P<0.01$ | |
| First live birth | | | | |
| No use | 256 | 624 | 1.00 | |
| Before 1st live birth | 9 | 30 | 0.60 | (0.24, 1.50) |
| Only after 1st live birth | 209 | 524 | 1.06 | (0.83, 1.35) |
| Users without live birth | 16 | 44 | 0.77 | (0.40, 1.45) |
| Unknown | 0 | 1 | | |

[a]Adjusted for age and use of other types of oral contraceptives; [b]Test for trend based on exposed subjects.

was observed in association with chlormadinone-containing combined products.

The joint effect of combined OCs with and without chlormadinone on risk was considered, and the estimate of the risk relative to non-users of either type was not greater for women who ever used both types (RR = 0.97; 95% CI = 0.72, 1.13) than for women who used only combined products with chlormadinone (RR = 0.90; 95% CI = 0.62; 1.29) or for those who only used combined products without chlormadinone (RR = 1.01; 95% CI = 0.73; 1.41).

### Discussion

Progestogens that are derivatives of 17-hydroxyprogesterone include medroxyprogesterone acetate and chlormadinone acetate. These compounds were shown in some studies to cause benign and malignant tumours in beagle dogs. Although both the results of these experiments and their relevance for human breast cancer have been questioned, these products were consequently withdrawn from public use as constituents of OCs in most countries (Vallance & Capel-Edwards, 1971; Giles *et al.*, 1978; Kwaplen *et al.*, 1980; El Etreby *et al.*, 1979, El Etreby & Neumann, 1980; Diszfalusy, 1982).

Most combined OCs contain progestogens that are 19-nortestosterone derivatives, and these compounds are likely the constituents that are responsible for the enhanced risk of thromboembolic phenomenon in users of OCs. Progestogens that are derived from 17-hydroxyprogesterone may not result in such adverse cardiovascular effects. If the influence of OCs that contain these compounds on risks of cancers in humans is not different or more favourable, than those associated with OCs that contain 19-nor-testosterone derivatives, then it would seem prudent to consider reintroducing such formulations for human use. Unfortunately, little information is available on the influence of such products on cancer risks in humans.

From 1966 to 1970, the only combined OC available in the GDR was a product that contained 3.0 mg chlormadinone and 100 μg mestranol. A combined product containing 2.0 mg chlormadinone and 80 μg mestranol was introduced in 1971. A sequential product containing 2.0 mg chlormadinone (taken for 7 days) and 100 μg mestranol (taken for 21 days) was introduced in 1970. Combined products without chlormadinone were first marketed in 1971, and sequentials without chlormadinone were first made available in 1976. From 1970 to 1978, the proportion of users who used products with chlormadinone decreased from 100% to 20% (Nischan & Ebeling, 1984). This variation in exposure provided a unique opportunity to investigate possible relationships between breast cancer and combined OCs with and without chlormadinone as the constituent progestogen.

The results from this study are quite likely to be valid. Selection bias is unlikely to have been a problem because few eligible cases or controls were not interviewed. Recall bias was minimised by selection of hospitalised controls, and by interviewing all study subjects in hospital. In addition, information on use of OCs that was obtained from interviews was supplemented by reviewing medical records for 80% of the women; and this proportion was similar for cases and controls, and for users of various types of OCs. The identification of associations between breast cancer and most of the generally accepted risk factors for this disease provides further reassurance as to the quality of the data. Furthermore, this study revealed no increased overall risk of breast cancer associated with use of all types of OCs combined, or of combined OCs without chlormadinone. This is in accordance with the findings from most recent investigations that have not been confined to specific ages or other subgroups of subjects (Lipnick *et al.*, 1986; Ellery *et al.*, 1986; Schlesselman *et al.*, 1988; La Vecchia *et al.*, 1986; Paul *et al.*, 1986; Rosenberg *et al.*, 1984; Vessey *et al.*, 1983; Brinton *et al.*, 1982).

Since the main subject of this investigation was the possible relationship between use of combined OCs with chlormadinone and breast cancer, separate analyses were performed to allow a comparison of results for products with and without this progestational agent. They showed that breast cancer risk did not increase with duration of use of combined OCs with or without chlormadinone. For either of these products, risk also did not change with time since initial exposure or with time since most recent use. However, there appeared to be an increase in risk in current users of both types of combined products. These findings, if not spurious, would be consistent with a promotional effect of OCs on the development of breast cancer.

The increase in risk for current users could, however, have resulted from a higher rate of screening for breast cancer among women who are users of OCs. Evidence against this explanation is the observation that the enhanced risk in recent users were observed for all sizes of tumour at diagnosis. On the other hand, routine mammographic examination with a higher detection rate of smaller tumours, had not been widely performed during the study period, and another study conducted in the GDR showed no statistically significant differences between size of tumours diagnosed by chance and by regular physical examination or regular monthly breast self examination (Kloskowski & Ebeling, 1990*a*; 1990*b*). Therefore, the fact that the enhanced risk could be observed for all sizes of tumours is not strong evidence against the observed associations being a result of more frequent breast examinations of users than non-users. In fact, in the GDR, guidelines of the Association of Obstetrics and Gynaecology advised all gynaecologists prescribing OCs to perform regular annual physical breast examinations on all users. Additionally, a higher proportion of cases than controls who were current users of combined OCs had recently started their use. Therefore, a screening effect seems to be a more likely explanation for the observed increase in risk in recent users than a causal relationship between recent use and breast cancer.

In conclusion, our investigation showed that chlormadinone as a constituent of combined OCs does not influence breast cancer risk.

This research received financial support from the Special Programme of Research, Development and Research Training in Human Reproduction, World Health Organisation.

### References

BRESLOW, N.E. & DAY, N.E. (1980). *Statistical Methods in Cancer Research*. Vol. 1: The analysis of case-control studies. IARC: Lyon (IARC Scient. Publ. No. 32).

BRINTON, L.A., HOOVER, R., SZKLO, M. & FRAUMENI, J.F. (1982). Oral contraceptives and breast cancer. *Int. J. Epidemiol.*, **11**, 316.

DICZFALUSY, E. (1982). Gregory Pincus and steroidal contraception revisited. *Acta Obstet. Gynecol. Scand. Suppl.*, **105**, 7.

EL ETREBY, M.F., GRAF, K.-J., BEIER, S., ELGER, W., GUNZEL, P. & NEUMANN, F. (1979). Suitability of the Beagle dog as a test model for the tumorigenic potential of contraceptive steroids. A short review. *Contraception*, **20**, 237.

EL ETREBY, M.F. & NEUMANN, F. (1980). Influence of sex steroids and steroid antagonists on hormone dependent tumours in experimental animals. In S. Iacobelli *et al.* (eds): *Hormone and Cancer*. New York: Raven Press, pp. 321–336.

ELLERY, C., MACLENNAN, R., BERRY, G. & SHEARMAN, R.P. (1986). A case-control study of breast cancer in relation to the use of steroid contraceptive agents. *Med. J. Aust.*, **144**, 173.

GILES, R.C., KWAPLEN, R.P., GEIL, R.G. & CASEY, H.W. (1978). Mammary nodules in Beagle dogs administered investigational oral contraceptive steroids. *J. Natl Cancer Inst.*, **60**, 1351.

INTERNATIONAL AGENCY FOR RESEARCH ON CANCER (1979). Chlormadinone acetate. In *IARC Monographs on the Evaluation of the Carcinogenic risk of Chemicals to Humans*. Vol. **21**. IARC: Lyon, pp. 365–375.

KLOSKOWSKI, S. & EBELING, K. (1990*a*). Zum Nutzen der jährlichen ärztlichen Untersuchung der Brust für die Früherkennung des Mammakarzinoms. *Z. Ärztl. Fortbild.*, **84**, 453.

KLOSKOWSKI, S. & EBELING, K. (1990*b*). Zum Nutzen der monatlichen Selbstuntersuchung der Brust für die Früherkennung des Mammakarzinoms. *Arch. Geschwulstforsch.*, **60**, 5.

KWAPLEN, R.P., GILES, R.C., GEIL, R.G. & CASEY, H.W. (1980). Malignant mammary tumors in Beagle dogs dosed with investigational oral contraceptive steroids. *J. Natl Cancer Inst.*, **65**, 137.

LA VECCIA, C., DECARLI, A., FASOLI, M. & 5 others (1986). Oral contraceptives and cancers of the breast and of the female genital tract. Interim results from a case-control study. *Br. J. Cancer*, **54**, 311.

LIPNICK, R.J., BURING, J.E., HENNEKENS, C.H. & 7 others (1986). Oral contraceptives and breast cancer. *J. Am. Med. Assoc.*, **255**, 58.

MCPHERSON, K., VESSEY, M.P., NEIL, A., DOLL, R., JONES, L. & ROBERTS, M. (1987). Early oral contraceptive use and breast cancer: Results of another case-control study. *Br. J. Cancer*, **56**, 653.

NELSON, L.W., CARLTON, W.W. & WEIKEL, J.H. (1972). Canine mammary neoplasms and progestogens. *J. Am. Med. Assoc.*, **219**, 1601.

NELSON, L.W., WEIKEL, J.H. & RENO, F.E. (1973). Mammary nodules in dogs during four year's treatment with megestrol acetate or chlormadinone acetate. *J. Natl Cancer Inst.*, **51**, 1303.

NELSON, L.W. & KELLY, W.A. (1976). Progestogen-related gross and microscopic changes in female Beagles. *Vet. Pathol.*, **13**, 143.

NISCHAN, P. & EBELING, K. (1984). Oral contraceptives containing chlormadinone acetate and cancer incidence at selected sites in the German Democratic Republic. A correlation analysis. *Int. J. Cancer*, **34**, 671.

PAUL, C., SKEGG, D.C.G., SPEARS, G.F.S. & KALDOR, J.M. (1986). Oral contraceptives and breast cancer: A national study. *Br. Med. J.*, **293**, 723T.

PIKE, M.C., HENDERSON, B.E., KRAILO, M.D., DUKE, A. & ROY, S. (1983). Breast cancer in young women and use of oral contraceptives: Possible modifying effect of formulation and age at use. *Lancet*, **ii**, 926.

PRENTICE, R.L. & THOMAS, D.B. (1987). On the epidemiology of oral contraceptives and disease. *Adv. Cancer Res.*, **49**, 285.

ROSENBERG, L., MILLER, D.R., KAUFMAN, D.W. & 4 others (1984). Breast cancer and oral contraceptive use. *Am. J. Epidemiol.*, **119**, 167.

SCHLESSELMAN, J.J., STADEL, B.V., MURRARY, P. & LAI, S. (1988). Breast cancer in relation to early use of oral contraceptives. *J. Am. Med. Assoc.*, **259**, 1828.

VALLANCE, D.K. & CAPEL-EDWARDS, K. (1971). Chlormadinone and mammary nodules. *Br. Med. J.*, **24**, 221.

VESSEY, M., BARON, J., DOLL, R., MCPHERSON, K. & YEATES, D. (1983). Oral contraceptives and breast cancer: Final report of an epidemiological study. *Br. J. Cancer*, **47**, 455.

WHO COLLABORATIVE STUDY OF NEOPLASIA AND STEROID CONTRACEPTIVES (1985). Invasive cervical cancer and combined oral contraceptives. *Br. Med. J.*, **290**, 961.

WHO COLLABORATIVE STUDY OF NEOPLASIA AND STEROID CONTRACEPTIVES (1990). Breast cancer and combined oral contraceptives: results from a multinational study. *Br. J. Cancer*, **61**, 110.

WORLD HEALTH ORGANISATION (1981). Histological typing of breast tumors, 2nd edn. WHO: Geneva.